# Microbial communities in the phyllosphere and endosphere of Norway spruce under attack by *Heterobasidion*

**DOI:** 10.3389/fmicb.2024.1489900

**Published:** 2025-01-08

**Authors:** Wen-jing Meng, Zi-lan Wen, Risto Kasanen, Hui Sun, Fred O. Asiegbu

**Affiliations:** ^1^Department of Forest Sciences, Faculty of Agriculture and Forestry, University of Helsinki, Helsinki, Finland; ^2^Collaborative Innovation Center of Sustainable Forestry in Southern China, College of Forestry, Nanjing Forestry University, Nanjing, China

**Keywords:** microbial community structure, *Heterobasidion*, network analysis, microbial interaction, microbial community diversity

## Abstract

*Heterobasidion annosum* species complex has been regarded as the most destructive disease agent of conifer trees in boreal forests. Tree microbiome can regulate the plant–pathogen interactions by influencing both host resistance and pathogen virulence. Such information would help to improve the future health of forests and explore strategies to enhance ecosystem stability. In this study, using next-generation sequencing technology, we investigated the microbial community in different tree regions (needles, upper stem, and lower stem) of Norway spruce with and without wood decay symptoms. The primary purpose was to uncover signature characteristic microbiome harbored by asymptomatic trees compared to diseased trees. Additionally, the study was to explore the inter-kingdom and intra-kingdom interactions in microbiome (bacteria and fungi) of symptomatic versus asymptomatic trees. The results showed that in upper stem, species richness (Chao1) of fungi and bacteria were both higher in asymptomatic trees than symptomatic trees (*P* < 0.05). Compared to symptomatic trees, asymptomatic trees harbored a higher abundance of Actinobacteriota, bacterial genera of *Methylocella*, *Conexibacter*, *Jatrophihabitans*, and fungal genera of *Mollisia*. Fungal communities from the same anatomic region differed between the symptomatic and asymptomatic trees. Bacterial communities from the two stem regions were also distinct between the symptomatic and asymptomatic trees. The symptomatic trees possessed a less stable microbial network with more positive correlations compared to the asymptomatic trees. In the lower stem, at intra-kingdom level, the distribution of correlation numbers was more even in the bacterial network compared to the fungal network. In conclusion, the *Heterobasidion* attack decreased the microbial community species richness and shifted the community structure and functional structure to varying degrees. The microbial network was enlarged and became more unstable at both inter-kingdom and intra-kingdom level due to the *Heterobasidion* infection.

## Introduction

Forest ecosystems act as one of the most powerful regulators in global climate change yet remain exposed to increasing biotic and abiotic challenges nowadays, including the carbon dioxide increase, warming, drought, and disease attack (Baldrian et al., 2023). In response to these problems, with the mediation of microbiome associations, forest ecosystems could therefore adapt to new environments (Addison et al., 2024). Microbes are ubiquitous and mainly resident in the phyllosphere, rhizosphere, and the internal tissues of plants (Xiong et al., 2023). As the communities associated intimately with plants, microbiome has the potential to act beneficially, commensally, and pathogenically on their host (Chialva et al., 2022).

The complex plant–microbiome interactions provide advantages to the plant host by promoting plant growth, assisting in nutrient uptake, increasing stress tolerance and resisting plant pathogens (Trivedi et al., 2020). In the field of resistance biology, plant microbiome potentially regulates the plant’s immune system and influence the pathogenic colonization as well as pathogenicity, ultimately modulate the whole plant–pathogen interaction (Xiong et al., 2023). Phyllosphere microbiome employs various strategies including competitive exclusion, antibiosis, induction of host immune response to exercise control over the pathogens (Bashir et al., 2022). For example, the phyllosphere bacterium *Methylobacterium* was shown to facilitate IAA mediated defense response against rot pathogens *Aspergillus niger* in groundnut (*Arachis hypogaea*) (Madhaiyan et al., 2006). Endophytes, as an important component of tree microbiome, were reported to suppress pathogens by directly competing with them for resources or by indirectly producing metabolites as antimicrobials (e.g., flavonoids, alkaloids, phenols, and peptides; Pańka et al., 2013; Pavithra et al., 2019). Furthermore, endophytes enhance plant growth through the biosynthesis of advantageous secondary metabolites, such as phytohormones, and by facilitating nutrient acquisition, including essential elements like phosphorus (Terhonen et al., 2018). Another example of microbiome facilitating in the suppression of pathogenic colonization is that the *Ampelomyces* spp. acting as intracellular parasites, could suppress the sporulation of powdery mildew fungi and kill the host fungal cells (Kiss, 2003). Some endophytic bacteria exhibit the ability to promote the growth of the plant by nitrogen fixation or plant hormone production, for example, *Azospirillum, Bacillus subtilis*, and *Burkholderia vietnamiensis* (Steenhoudt and Vanderleyden, 2000; Xin et al., 2009; Hashem et al., 2019), mediating the plant–pathogen interaction in an indirect way.

On the other hand, plant-associated microbial community assembly could be shaped by various factors during the host–pathogen interaction. The leaf epiphytic bacterial species richness was found to decrease when southern leaf blight disease severity increased (Manching et al., 2014). The endophytic bacterial community structures also shifted in response to methyl bacterium-induced protection of plants against pathogens (Ardanov et al., 2012).

The root and butt rot on conifer trees caused by *Heterobasidion annosum* species complex was considered the most devastating fungal diseases in the northern hemisphere (Asiegbu et al., 2005; Garbelotto and Gonthier, 2013). In Europe, the annual financial loss caused by the species complex was estimated around 800 million euros due to the decay as well as overall reduction in the diameter growth of diseased trees (Asiegbu et al., 2005; Hellgren and Stenlid, 1995; Zeng et al., 2018). Distinct host preference was shown among the three Eurasian intersterile groups of *H. annosum* species complex, *H. annosum* s.s. usually attacks pines (*Pinus* spp.), while *Heterobasidion parviporum* preferentially infects Norway spruce [*Picea abies* (L.) Karst.], and *Heterobasidion abietinum* is commonly associated with silver fir (*Abies alba* Mill.) (Garbelotto and Gonthier, 2013). *H. annosum* s.l. primarily uses the basidiospores to infect freshly open wood surfaces and then spread to neighboring hosts by root to root contact (Zaļuma et al., 2019). For decades, the *Heterobasidion* root rot has been managed by use of the fungal biocontrol agent *Phlebiopsis gigantea* (Fr.) Julichto which is able to outcompete and out-grow *Heterobasidion* on stump surfaces (Pratt et al., 2000; Kärhä et al., 2018). Although fewer bacterial biocontrol cases against forest fungal pathogens were reported than those against crop fungal pathogens (Singh et al., 2020), bacteria isolated from healthy *Pinus radiata* rhizosphere were reported to inhibit the growth of *H. annosum in vitro* and also protect *P. radiata* seedlings (Mesanza et al., 2016). Apart from these antagonistic screening studies, with the help of next-generation sequencing, recent studies have focused on the host microbial communities during *Heterobasidion* infection to further explore the complicated interactions among the microbiome, pathogen, and host. The fungal and bacterial community associated with different anatomic regions (root, bark, down stem, upper stem, and needles) of Norway spruce trees with and without wood decay symptoms were compared respectively to understand the impact of *Heterobasidion* infection (Kovalchuk et al., 2018; Ren et al., 2019). The microbial community inhabiting *Heterobasidion* fruiting body and adhering dead wood at different decay stages were also studied, revealing the infection process at a “dynamic” level (Ren et al., 2022, 2023).

Though many studies have focused on microbial communities, not much attention is given to the interactions of microorganisms within the community *in situ*. However, more evidence showed that priority should be given to microbial network shifts because it could unravel their functions and vulnerability to various disturbances (de Vries et al., 2018). Analyzing microbial networks and identifying core microbes are crucial for understanding the factors that contribute to microbial resilience when subjected to biotic or abiotic disturbances (Astudillo-García et al., 2017; Lemanceau et al., 2017). This analysis also provides opportunity to find promising antagonistic agents against *Heterobasidion*, since competing relationships with the fungal pathogen could be indicated by the negative correlations between them (Ren et al., 2023). Using co-occurrence microbial network analysis, conclusions were drawn that showed that compared to soil fungal networks, soil bacterial networks represented less stability under drought and linked more tightly to soil functioning during recovery (de Vries et al., 2018). Another study revealed that drought disrupted microbial networks both among bacteria, among fungi, and between bacteria and fungi significantly (Gao et al., 2022).

In this study, a combined taxonomic analysis and network analysis of the phyllosphere and endospheric microbiomes from different regions of Norway spruce trees were conducted. The objectives were to (i) uncover signature characteristic microbiome harbored by asymptomatic trees compared to diseased trees and to (ii) explore the network interaction in microbial community of asymptomatic versus symptomatic *Heterobasidion* decayed trees.

## Materials and methods

### Study sites and sample collection

The sampling site was a Norway spruce [*P. abies* (L.) Karst.] dominated forest with a relatively high incidence of *Heterobasidion* infection in Myrskylä (Uusimaa region, Southern Finland). The spruce forest is privately owned and targeted for commercial timber production. The spruce trees at the designated locations had undergone natural regeneration and were of the same age around 62 years.

**FIGURE 1:**
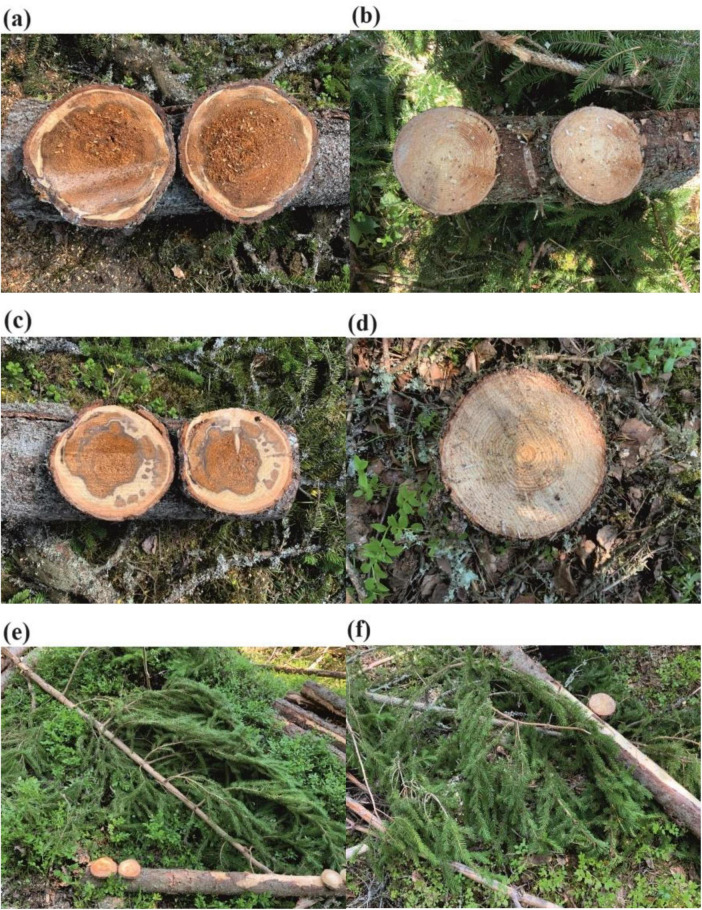
The lower stems (stems at stump base height) **(a)**, upper stems (stems at breast height) **(c)**, needles **(e)** from symptomatic Norway spruce and the lower stems **(b)**, upper stems **(d)**, needles **(f)** from asymptomatic Norway spruce.

In June 2023, sample collection was carried on trees which were cut down during harvesting. In this way, decayed trees could be distinguished from healthy ones based on the brown stains which represented wood decay symptoms on the stump surfaces (Figure 1). Finally, we sampled 10 decayed trees and 5 healthy trees from the four plots which were located very close to each other: (1) 60°40′04.47″N, 25°45′49.62″E, (2) 60°40′02.78″N, 25°45′49.25″E, (3) 60°40′02.94″N, 25°45′49.07″E, (4) 60°40′05.45″N, 25°45′50.34″E. Needles and stems at both stump base height and breast height from each sampled tree were collected.

Once collected, the samples from each sample tree were shipped with ice pack and stored at −20°C for later laboratory processing. For the group names described further in the text and figures, the first letter S and A stand for symptomatic trees and asymptomatic trees, respectively. The second letter L, U, and N stands for lower stems (stems at stump base height), upper stems (stems at breast height), and needles, respectively.

### DNA extraction, amplification of fungal ITS2, bacterial V3–V4 region, and sequencing

Due to the enormous size of each stem, we firstly cut a wood piece which contained heartwood, sapwood and bark from the stem and then split this piece into small cubes with aid of sterilized tools. The wood cubes from the same stem were then homogenized using mixer mill MM400 (Retsch, Germany) following the manufacturer’s instruction. Needles were homogenized manually with mortars and pestles. The standard cetyltrimethylammonium bromide (CTAB) method (Chang et al., 1993) with modifications (Terhonen et al., 2011) was followed to extract the DNA from samples. NanoDrop ND-1000 spectrophotometer (Thermo Fisher Scientific, USA) was used to measure the quality and concentration of the isolated DNA.

PCR amplification of the fungal ITS2 region and bacterial V3–V4 region and sequencing were performed at Novogene (Cambridge Science Park, UK). The fungal and bacterial amplification was performed using primers ITS3-2024F (5′-GCATCGATGAAGAACGCAGC-3′) and ITS4-2409R (5′-TCCTCCGCTTATTGATATGC-3′) with primers 341F (5′-CCTAYGGGRBGCASCAG-3′) and 806R (5′-GGACTACNNGGGTATCTAAT-3′), respectively. Raw sequences are available from the Sequence Read Archive (SRA) on National Center for Biotechnology Information (NCBI) under project number PRJNA1107117 for bacteria and under project number PRJNA1107041 for fungi.

### Sequence processing and data analyses

Paired-end reads were first assigned to samples and truncated by cutting off their unique barcode and primer sequences. Raw tags were generated after merging the paired-end reads using FLASH (V1.2.1.11^1^) (Magoč and Salzberg, 2011). Quality filtering on the raw tags went on using the fastp (Version 0.23.1) software to obtain high-quality clean tags (Bokulich et al., 2013). Based on reference database [Silva database (16S/18S)^2^ ; Unite database (ITS)^3^), chimera sequences were detected and removed with the vsearch package (V2.16.0^4^) (Edgar et al., 2011), therefore generated the effective tags. Then denoise was performed with DADA2 in the QIIME2 software (Version QIIME2-202202) to obtain initial ASVs (Amplicon Sequence Variants) which were annotated to species afterward in the same software using Silva database for 16S/18S sequences or United database for ITS sequences. The absolute abundance of ASVs was normalized using a standard of sequence number corresponding to the sample with the least sequences. Subsequent analysis of alpha diversity was performed based on the output of normalized data. Three alpha diversity indices including Chao1, Simpson’s Index of Diversity (1-D), and Pielou’s evenness were selected to reflect the microbial community species richness, microbial community diversity, and microbial community evenness, respectively. In SPSS v26.0 (Chicago, IL, USA), one-way analysis of variance (ANOVA) was adopted to examine the significant differences of the alpha diversity indices while independent-samples *T*-test was used to examine the significant differences of various parameters between asymptomatic trees and symptomatic trees. GraphPad Prism 8 was adopted to generate the figures.

We performed the network analysis using ASVs with relative abundance over 0.1% (Ren et al., 2023). The Spearman correlations with correlation value over 0.8 and *P*-value lower than 0.05 between ASVs were calculated and filtered in R. The keystone ASVs were further selected with the top 10% abundance in the network to do the keystone analysis. Then the visualization was done by Gephi (Version 0.9.2) based on the correlation results. The degree distribution and number of edges were calculated in Gephi and visualized further in R.

## Results

### Microbial community alpha diversity

**FIGURE 2:**
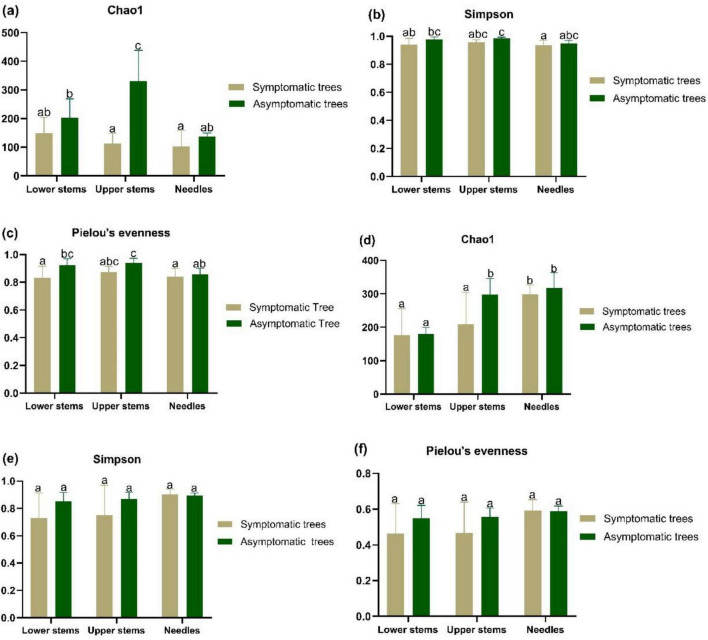
The alpha diversity bacterial community **(a–c)** and fungal community **(d–f)** in symptomatic and asymptomatic trees. Bacterial community species richness (Chao1) **(a)**, diversity (Simpson’s diversity) **(b)**, and evenness (Pielou’s evenness) **(c)** of community. Fungal community species richness (Chao1) **(d)**, diversity (Simpson’s diversity) **(e)**, and evenness (Pielou’s evenness) **(f)**. Letters above the column represent the significant difference (*P* < 0.05) among groups.

In upper stems, the species richness of both bacterial community and fungal community was higher in asymptomatic trees than symptomatic trees (*P* < 0.05) (Figures 2a, d). Additionally, the highest species richness of bacterial community from asymptomatic trees was observed in upper stems compared to needles and lower stems (*P* < 0.05) (Figure 2a). The highest species richness of fungal community from symptomatic trees was observed in needles, while the fungal community in upper stem and needles from asymptomatic trees exhibited higher species richness than the lower stem (*P* < 0.05) (Figure 2d). No significant difference of Simpson’s diversity or Pielou’s evenness was found between symptomatic trees and asymptomatic trees (Figures 2b, c, e, f).

### Microbial community composition and structure

**FIGURE 3:**
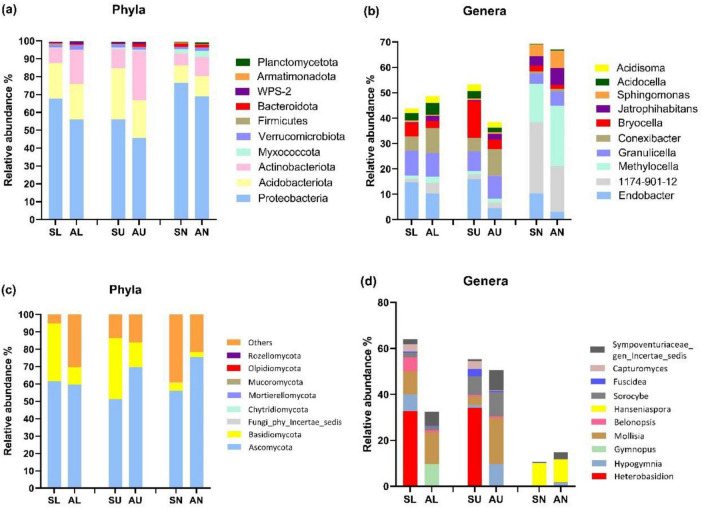
The top 10 most abundant microbial taxa at phylum and genus level in the three regions from symptomatic and asymptomatic *Picea abies* tree. The bacterial phyla **(a)**, bacterial genera **(b)**, fungal phyla **(c)**, and fungal genera **(d)**. The first letter S and A stand for symptomatic trees and asymptomatic trees, respectively, while the second letter L, U, and N stand for lower stems (stems at stump base height), upper stems (stems at breast height), and needles.

Proteobacteria, Acidobacteriota, and Actinobacteriota emerged as the predominant bacterial phyla across all phyla (Figure 3a). Additionally, symptomatic trees exhibited a higher abundance of Proteobacteria and a lower abundance of Actinobacteriota compared to asymptomatic trees (Figure 3a). At bacterial genus level, *Endobacter*, *Bryocella* were more abundant in the symptomatic trees while *Methylocella*, *Conexibacter*, and *Jatrophihabitans* were more abundant in the asymptomatic trees (Figure 3b). Ascomycota and Basidiomycota dominated fungal community, and symptomatic trees exhibited higher abundance of Basidiomycota especially in stem regions while asymptomatic trees had more abundant Ascomycota in upper stem and needles (Figure 3c). Genus *Heterobasidion* was found to be prevalent in symptomatic lower stem (32.6%) and symptomatic upper stem (34.2%), and also present rather infrequently in symptomatic needles (0.07%), asymptomatic lower stem (0.002%), and asymptomatic upper stem (0.004%) (Figure 3d). *Mollisia* showed more frequently in asymptomatic trees while *Fuscidea* and *Capturomyces* exhibited higher abundance along with *Heterobasidion* in symptomatic trees (Figure 3d).

Based on *T*-test, the bacterial species *Actinomycetales bacterium* and *Acidobacteria bacterium* CU2 were more abundant in the upper stem of asymptomatic trees than those of symptomatic trees (*P* < 0.05) (Table 1). Fungal species *Cyphellophora sessilis* and *Fellomyces horovitziae* were more abundant in the needles from asymptomatic trees than those from symptomatic trees (*P* < 0.05) (Table 1).

**TABLE 1:** The microbial species among the top 50 most abundant fungal and bacterial species showing significant differences in higher relative abundance between asymptomatic and symptomatic trees based on *T*-test.

Anatomic regions	Species	*P*
Upper stems	*Actinomycetales bacterium*	0.024
	*Acidobacteria bacterium* CU2	0.013
Needles	*Cyphellophora sessilis*	0.004
	*Fellomyces horovitziae*	0.043

**FIGURE 4:**
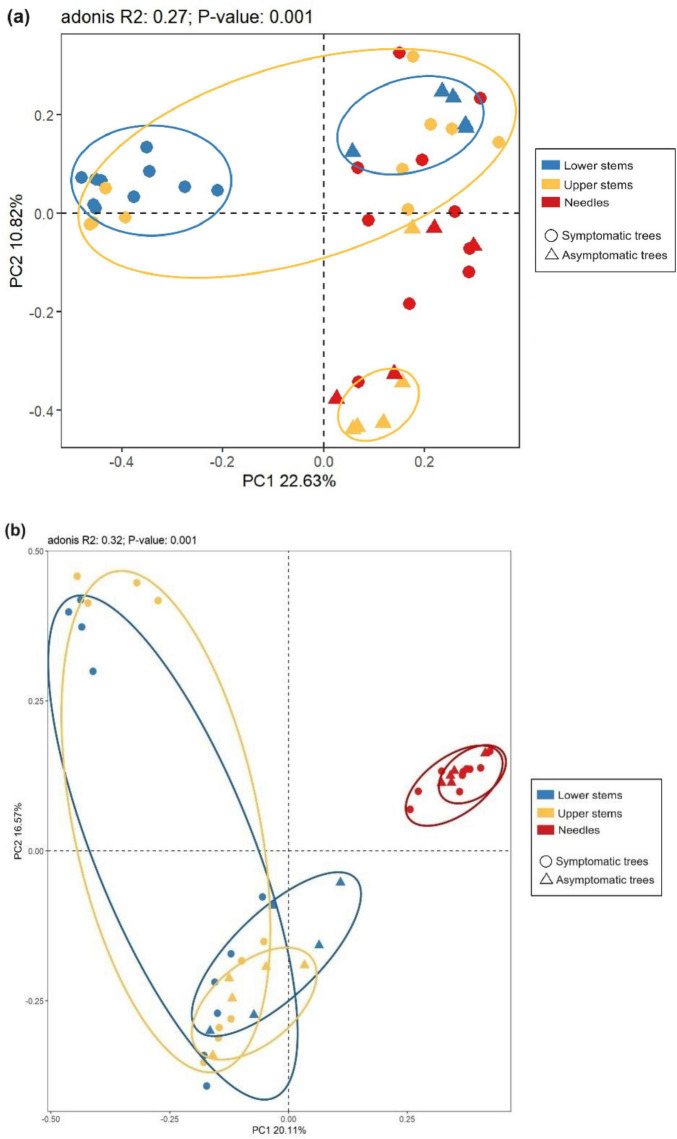
Principal co-ordinates analysis (PCoA) illustrates microbial community structures. Bacterial community structures **(a)** and fungal community structures **(b)**. The colorful eclipses represent the separation of microbial community from the same region between symptomatic and asymptomatic trees.

The principal co-ordinate analysis (PCoA) and subsequent permutational multivariate analysis of variance (PERMANOVA) revealed that the bacterial community in upper stems and lower stems differed between symptomatic trees and asymptomatic trees, respectively (*P* < 0.05) (Figure 4a). The fungal communities in the same region were distinct between symptomatic and asymptomatic trees (*P* < 0.05) (Figure 4b). The lower stem formed distinct bacterial community with the other two regions in both symptomatic and asymptomatic trees (*P* < 0.05) (Figure 4a). In symptomatic trees, the needles form distinct fungal community with the other two regions (*P* < 0.05) while each region from asymptomatic trees harbored distinct fungal community (*P* < 0.05) (Figure 4b).

### Predicted microbial community functional structures

**FIGURE 5:**
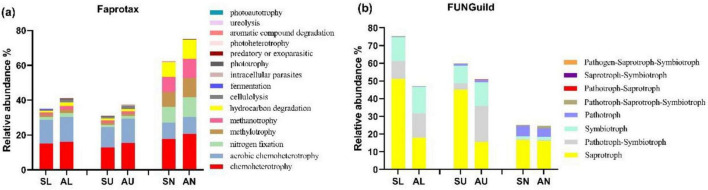
The relative abundance of the top 15 microbial trophic modes based on faprotax analysis for bacteria and FUNGuild analysis for fungi. Bacterial trophic modes **(a)** based on faprotax analysis, and the fungal trophic modes **(b)** based on FUNGuild analysis, respectively. For the group names, the first letter S and A stand for symptomatic trees and asymptomatic trees, respectively, while the second letter L, U, and N stand for lower stems (stems at stump base height), upper stems (stems at breast height), and needles.

Chemoheterotroph (15.55%) was the most abundant bacterial functional modes in all samples, followed by aerobic chemoheterotroph (11.81%), nitrogen fixation (4.11%), hydrocarbon degradation (3.69%), and methylotrophy (3.68%) (Figure 5a). The bacterial trophic mode of intracellular parasites in needles from symptomatic trees outnumbered that from asymptomatic trees (*P* < 0.05) (Figure 5a). The composition of bacterial functional modes was quite similar in the stem regions, while the needles seemed to have a different composition pattern which was more even (Figure 5a). Saprotroph (30.59%) dominated in the fungal trophic modes, followed by symbiotroph (8.87%), pathotroph-symbiotroph (7.05%), and pathotroph (2.28%) (Figure 5a). The abundance of saprotroph mode was more abundant in the lower and upper stems from symptomatic trees than that from asymptomatic trees (*P* < 0.05) (Figure 5b).

**FIGURE 6:**
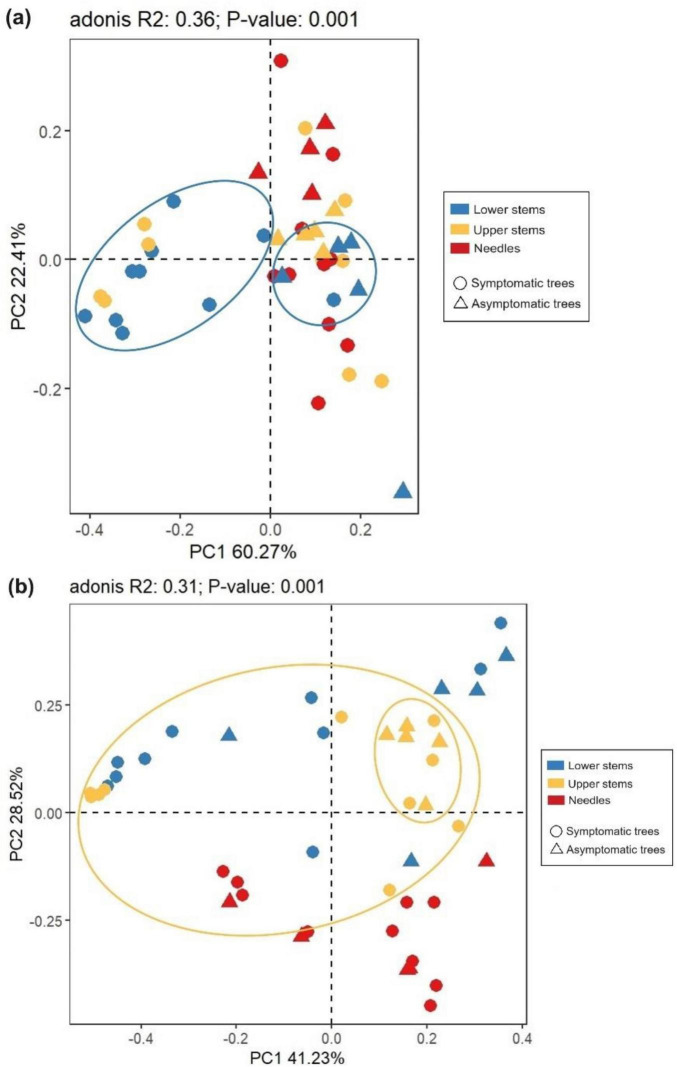
Principal co-ordinates analysis (PCoA) illustrating the microbial community functional structures. Bacterial community functional structures **(a)** and fungal community functional structures **(b)**. The colorful eclipses represent the separation of microbial community from the same region between symptomatic and asymptomatic trees.

Functionally, symptomatic trees formed distinct bacterial community with asymptomatic trees in lower stem, and distinct fungal community with asymptomatic trees in upper stem, respectively (*P* < 0.05) (Figures 6a, b). On symptomatic trees, bacterial functional community in lower stem separated with other two regions (*P* < 0.05) while each region from asymptomatic trees formed their own bacterial functional community structure (*P* < 0.05) (Figure 6a). As to fungal functional community, both symptomatic trees and asymptomatic trees, needles formed distinct structures with the other two regions (*P* < 0.05) (Figure 6b).

### Microbial inter-kingdom and intra-kingdom network analysis

**FIGURE 7:**
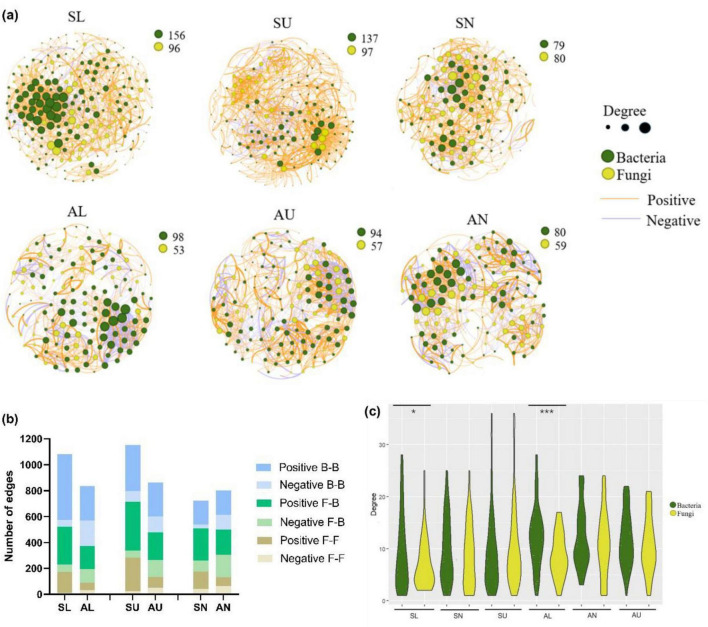
Microbial network analysis in symptomatic trees and asymptomatic trees. Microbial co-occurring network in symptomatic trees and asymptomatic trees **(a)**. Each node represents a bacterial/fungal ASV in the network. The degree stands for the connection amount with a particular node, which is indicated by the node size. The thickness of the edge between two nodes represents their correlation level. The number of positive and negative edges within inter-kingdom and intra-kingdom network **(b)**. F-F, fungi to fungi; F-B, fungi to bacteria; B-B, bacteria to bacteria. The distribution of degrees within bacterial and fungal nodes **(c)**. The symbols ‘*’ and ‘***’ represent a significance difference (*P* < 0.05) and a significance difference (*P* < 0.001) respectively.

Except for the bacterial ASVs in needles, more microbial ASVs were present in the symptomatic trees than in the asymptomatic trees (Figure 7a). In total, a higher number of edges were observed in each region from symptomatic trees than from asymptomatic trees (Figures 7a, b). The two stem regions possessed higher numbers of ASVs and edges than the needles (Figures 7a, b). Positive edges (77.8%) were more abundant than negative edges in total (22.2%) (Figures 7a, b), however, higher portion of edges were assigned to positive group in the symptomatic trees compared to the asymptomatic trees (Figure 7b). For the intra-kingdom and inter-kingdom correlations, the fungi-fungi edges accounted for the lowest proportion in all regions, while fungi-bacteria edges accounted for the highest proportion in the needle region and bacteria-bacteria edges remained the most in both lower stem and upper stem regions (Figure 7b).

As to the distribution of degrees, bacterial ASVs appeared to exhibit more even degree pattern than fungal ASVs from the lower stem in both symptomatic trees and asymptomatic trees (*P* < 0.05) (Figure 7c).

**FIGURE 8:**
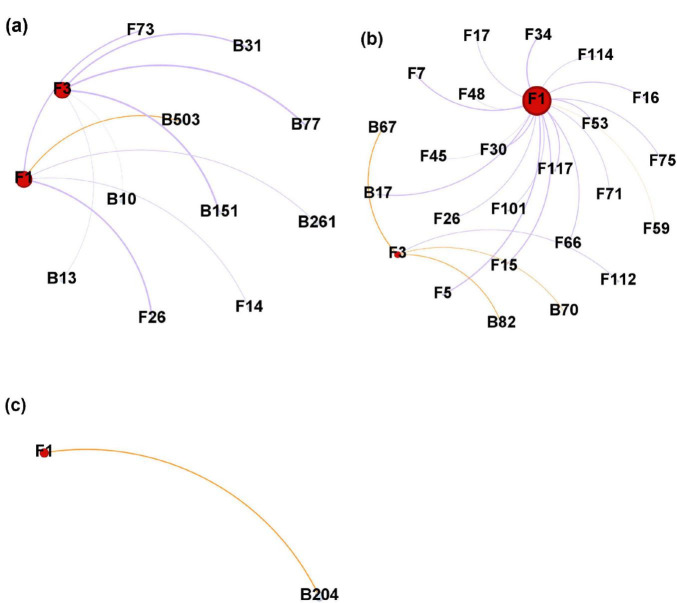
Microbial network generated from *Heterobasidion* ASVs and their connected ASVs in lower stems **(a)**, upper stems **(b)**, and needles **(c)** from symptom trees. B, bacterial ASV; F, fungal ASV. Orange edges present positive correlations, purple edges represent negative correlations. F1 and F3 are *H. parviporum* ASVs.

The negative correlations related to *Heterobasidion* ASVs occupied a higher ratio than the positive correlations in the two stem regions (Figures 8a, b), while only one positive correlation was formed with *Heterobasidion* ASVs in the needles (Figure 8c). Higher percentage of negative edges were built with bacterial ASVs in the lower stem, around 66.67% (Figure 8a). However, a higher percentage of negative edges were built with fungal ASVs in the upper stem, around 94.74% (Figure 8b). The upper stem harbored the largest microbial network connected to *Heterobasidion* ASVs among the three regions (Figures 8a–c).

Based on the network analysis, potential antagonistic fungal agents were proposed by the negative correlated ASVs (Table 2). One fungal ASV *Scoliciosporum umbrinum* was from the lower stem, while other three fungal ASVs including *Hypogymnia physodes*, *Fuscidea pusilla*, and *Lecidea nylanderi* came from the upper stem (Table 2).

**TABLE 2:** The ASVs among the top 10% most abundant ASV within the network showing negative correlation with *H. parviporum*.

ASVs	Correlation value	Region
*Scoliciosporum umbrinum*	−0.81818	Lower stem
*Hypogymnia physodes*	−0.87397	Upper stem
*Fuscidea pusilla*	−0.86293	Upper stem
*Lecidea nylanderi*	−0.78521	Upper stem

## Discussion

It is almost 150 years ago when the first book linking root and butt rot disease of conifers to *H. annosum* was published by Hartig (1874). Despite the length of time that has elapsed, this disease is still one of the most severe threats to conifer trees especially in Europe (Asiegbu et al., 2005). Consequently, it is still crucial for forest pathologists to keep on conducting and exploring research approaches for the management of the disease with the aid of updated modern tools and techniques. In this study, we investigated the microbial community in different anatomic regions of Norway spruce with and without wood decay symptoms to better understand the role of microbiomes in the tree–pathogen interaction.

Both bacterial and fungal community in the upper stem from the asymptomatic trees harbored higher species richness (Chao1) than those from the symptomatic trees. Other authors observed that that leaf bacterial alpha diversity (Chao1) was lower in pathogen-inoculated maize plots than those without pathogen inoculation (Manching et al., 2014). Soil bacterial species richness (Chao1) was reduced with the increasing severity of bacterial wilt disease caused by *Ralstonia* (Yang et al., 2017). Fungal alpha diversity of healthy tobacco leaves was also higher than that of diseased leaves (Sun et al., 2023). High proportion of *Heterobasidion* was found to be prevalent in symptomatic lower stem (32.6%) and symptomatic upper stem (34.2%), and also rather infrequently in symptomatic needles (0.07%), asymptomatic lower stem (0.002%), and asymptomatic upper stem (0.004%). With respect to the prevalence of *Heterobasidion* spp. in stem regions, it is quite normal because *Heterobasidion* spreads through root to root contact and up the tree trunk. Obvious decay symptoms would manifest in both lower stems and upper stems depending on the stage of the infection (Asiegbu et al., 2005). Additionally, part of the reason why there was more *Heterobasidion* in upper stem than lower stem was that in highly decayed lower stem, the host defense system may have been severely weakened consequently making it possible for other saprotrophic fungi to colonize leading to successional changes (Kovalchuk et al., 2018). The presence of *Heterobasidion* in the needles could be due to aerial spores within the vicinity of the sampled trees.

With respect to variations in the bacteria biota of the sample trees, the bacterial community in the lower stem was more even in the asymptomatic trees than that from symptomatic trees, indicating possible interference of *Heterobasidion* infection on the bacterial community. Other studies have reported that bacterial leaf blight increased the alpha diversity of bacterial community in rice leaves (Yang et al., 2020). By contrast, no significant change in microbial species richness was induced by the infection of *Septoria glycines* and *Phytophthora sojae* (Díaz-Cruz and Cassone, 2022). These results were also expected since the incidence of disease is likely to lead to weakness of host plants thereby increasing the chance for the colonization by other microorganisms (Yang et al., 2020).

Compared to symptomatic trees, asymptomatic trees exhibited a higher abundance of Actinobacteriota phylum which is well known for their outstanding ability to secret antibiotic compounds (Saxena, 2014; Boukhatem et al., 2022). It is most probable that symptomatic trees have reduced ability to harbor many of the antibiotic producing isolates that could have contributed in suppressing *Heterobasidion* infection. At the genus level, *Methylocella*, *Conexibacter*, and *Jatrophihabitans* were more abundant in asymptomatic trees. Among these genera, the genus *Methylocella* occupied a considerable proportion (17.69%) in the needles and belonged to the facultatively methanotrophic bacteria (Dedysh et al., 2005). However, in contrast to previous reports, a frequently low appearance of methanotrophs (below 1%) was detected in the phyllosphere bacterial community using metagenomic approaches (Hiroyuki et al., 2015). This genus is able to utilize multi-carbon compounds including acetate, pyruvate, succinate, etc. (Dedysh et al., 2005). The possible difference in abundance of these isolates between symptomatic needles and asymptomatic needles may suggest that the biosynthesis of these compounds was suppressed in *Heterobasidion* infected Norway spruce. The endophytic bacterial genus *Conexibacter* (Li et al., 2023), was also reported to be less abundant in Huanglongbing-infested rhizosphere of Citrus than healthy rhizosphere and correlated positively with the available potassium, available phosphorus and organic matter (Lei et al., 2024). It may be inferred that the *Heterobasidion* infection may have influenced the microbes leading to decrease in the abundance of this *Conexibacter* in the stem region. The abundance of *Heterobasidion* was found to be rather infrequent in needles (0.07%) than that in stem regions from symptomatic trees, indicating that the growth of *Heterobasidion* could barely reach the needles in the infected Norway spruce. Interestingly, *Heterobasidion* also showed up in stem regions from asymptomatic trees at a very low percentage (0.002% and 0.004% for lower stem and upper stem, respectively), demonstrating that even without wood decay symptom, Norway spruce trees in the field could have small amount of *Heterobasidion* inoculums. It is possible that infection is at an early stage or that the trees have partial resistance that delays manifestation of symptoms. It is also possible that the accumulation of this fungi have not reached a suitable amount to cause the decay or the asymptomatic trees were able to restrict the *Heterobasidion* growth by individual genetic resistance background as well as tree microbiome antagonistic agents (Kovalchuk et al., 2018).

In the two genera which showed more frequently in asymptomatic trees, *Mollisia* is a genus of frequently encountered saprotrophic fungi found on decaying plant tissues in temperate regions (Tanney and Seifert, 2020). Some endophytic species within this genus were reported to produce antifungal secondary metabolites (Fan et al., 2016; Tanney and Seifert, 2020). Furthermore, we could not explain the higher abundance of Endobacter and Bryocella in symptomatic than in asymptomatic trees. However, many Endobacteria have been found associated with ectomycorrhizal fungi whereas many members of Bryocella are commonly isolated from forest soil and methanotrophic environments (Bertaux et al., 2005; García-Fraile et al., 2016). This probably suggests they may have some functional relevance in the ecosystem which deserves to be explored.

Using network analysis, we explored possibility to identify potential biocontrol agents from healthy hosts (Mesanza et al., 2016). Based on *T*-test, and network analysis, we identified four potential antagonistic agents including fungal species *C. sessilis* and *F. horovitziae*, bacterial species *A. bacterium* and *A. bacterium* CU2. Among these, *A. bacterium* belongs to the order Actinomycetales which is known for producing antimicrobial compounds and could be a rich source of antibiotics (Genilloud, 2017; Takahashi and Nakashima, 2018). One Actinomycetales bacteria isolated from a spruce stand was reported to suppress the growth of *H. annosum* in the coculture experiments (Maier et al., 2004). Acidobacteria are abundant in soil and has potential to become beneficial rhizosphere microbes, but remain difficult to cultivate (Kalam et al., 2020). However, *C. sessilis* was reported to cause sooty blotch and flyspeck, an economically damaging disease of certain fruit crops, as well as epiphytic colonies on bamboo (Gao et al., 2015). *F. horovitziae* was reported as a basidiomycetous yeast species isolated from a *Xenasmatella* basidiocarp (Spaaij et al., 1991).

Between symptomatic trees and asymptomatic trees, bacterial communities differed in the two stem regions while fungal community was distinct in each region. Many studies also reported the endophytic or phyllosphere microbial community shift in response to different diseases (Bulgari et al., 2014; Luo et al., 2019 Yang et al., 2020; Díaz-Cruz and Cassone, 2022; Sun et al., 2023). In the tree–pathogen interaction, Norway spruce trees tend to develop the reaction zone between the decayed heartwood and undecayed sapwood where pH increases to neutrality or alkalinity due to the accumulation of carbonate and phenolic compounds (Shain, 1971; Johansson and Theander, 1974). In addition, inoculation with *H. parviporum* was found to activate the peroxidases production in Norway spruce trees (Liu M. et al., 2021). The physiochemical response in the stem region of symptomatic Norway spruce trees could explain the variation in microbial community structures. It is interesting that the shift in fungal community structure was observed in the needles. However, even though only two symptomatic trees had yellow needles when the samples were collected. It is possible that defense reactions provoked by the invading *Heterobasidion* pathogen Norway spruce may have had an influence on the water and nutrient transportation, including producing phenolic compounds in ray parenchyma cells, activating phloem parenchyma cells and forming traumatic resin ducts in the xylem (Krekling et al., 2004), and consequently induce the shift of fungal community in the needles. The bacterial community from the needles, unlike the fungal community, was not distinct between symptomatic and asymptomatic trees which might suggest that fungal communities are more sensitive to various perturbations including plant disease and moisture variation (Kaisermann et al., 2015; Liu L. et al., 2021). The fungal communities in the two stem regions from symptomatic trees did not differ with each other, which is different from the a previous report that each anatomic region formed a distinct fungal community (Kovalchuk et al., 2018). The differences in the observation could be related to variations in the infection level and stages of development of *Heterobasidion*. Lower stem formed distinct bacterial community in both symptomatic trees and asymptomatic trees, demonstrating that regardless of the *Heterobasidion* infection, upper stem and needles in Norway spruce shared similar bacterial communities together.

Chemoheterotrophy and aerobic chemoheterotrophy were the top two bacterial functional modes in the stem regions, same with the research in rhizosphere and soil bacterial community (Liu et al., 2022; Sun et al., 2022; Zhang et al., 2023). The dominance of these two modes in the stems indicated that most of the bacteria members were not able to fix carbon and the carbon sources were obtained as energy by the oxidation of organic compounds (Zhang et al., 2018). In needles, nitrogen fixation, methylotrophy, methanotrophy, and hydrocarbon degradation were abundant, demonstrating the ability of bacterial community dwelling in the needles to make use of carbon sources as well as nitrogen. This is likely due to the presence of methanotrophy and nitrogen fixing bacteria in the needles (Fürnkranz et al., 2008; Hiroyuki et al., 2015; Zhu et al., 2023). In the needles, the abundance of intracellular parasitic bacteria in symptomatic Norway spruce trees may have been enhanced by the deteriorating host health status and weakening immune response (Silva and Silva Pestana, 2013). It is however not surprising that the most abundant mode saprotroph was more common in symptomatic trees (Garbelotto and Gonthier, 2013). The invading *Heterobasidion* could also provide a more favorable living conditions for other saprotrophic fungi (Wen et al., 2019). Between symptomatic and asymptomatic trees, the bacterial functional structures differed only in lower stem. Fungal functional structures differ in upper stem, which is more restrained than microbial community differences. The reason could be that some microbial species share a common trophic mode. It is interesting that each region from symptomatic trees formed distinct bacterial functional structure, indicating that under the *Heterobasidion* infection, bacteria in different regions of Norway spruce tended to shift to diverse lifestyle modes.

Different from community composition, more complexity could be revealed in resistance of microbial communities by microbial network (Gao et al., 2022). In our study, the network analysis unveiled that the *Heterobasidion* infection enlarged and complicated the microbial network in the stem regions with more ASVs and correlations, which is in line with the previous study on tobacco rhizosphere and endosphere microbial network with and without wilt disease (Tan et al., 2021). The invasion of pathogens is likely to break the microbalance in the community, create more chances for the colonization of other species and generate more intense relationships (Hu et al., 2020). In addition, a larger proportion of negative correlations was observed in all regions from the asymptomatic trees, demonstrating the higher stability of microbial communities to a deeper level since the microbial competition could dampen cooperative networks and increase stability (Coyte et al., 2015; de Vries et al., 2018). In general, *Heterobasidion* infection influenced the microbial network at a profound level, by enlarging the network scale and breaking the network stability. Furthermore, it was possible to predict a more balanced microbial network in the needles by the domination of correlations between fungi and bacteria. This is partly because the communication between fungi and bacteria plays a key role in maintaining the balance of microbial community structure (Bamisile et al., 2018; Tan et al., 2021). In the lower stem, the distribution of degrees was more even in bacterial community than fungal community, which is in contrast to the previous study (Ren et al., 2023). The more even bacterial degree distribution was induced by the bacterial ASVs which stayed at higher connections, illustrating that bacterial members rather than fungal members played a decisive role in the network of the lower stem.

To further explore the pattern of network induced by *Heterobasidion* infection, we visualized the *Heterobasidion*-centered network. It was obvious that negative correlations dominated in the two stem regions, which clarifies the prevalence of antagonistic relationships built with *H. parviporum*. The host-associated microorganisms always establish competitive relationships with the pathogen, by nutrient competition, niche occupation, immune regulation, and modulation of virulence factors (Kavita and Howard, 2017; García-Bayona and Comstock, 2018). In addition, the bacteria and fungi played the vital role as antagonistic agents in the lower stem and upper stem, respectively.

The four potential antagonistic agents which were negatively correlated with *H. parviporum*, however, were four lichen species. *H. physodes* is a lichenized fungus which can produce secondary compounds with antibacterial activity as well as inhibitory effects on enzymes (Christian et al., 2008; Dymytrova, 2011; Lendemer, 2011; Studzinska-Sroka and Zarabska-Bozjewicz, 2023).

## Conclusion

Compared to symptomatic trees, asymptomatic trees harbored microbial community with higher species richness and evenness. Taxonomically, asymptomatic trees exhibited a higher abundance of Actinobacteriota, bacterial genera of *Methylocella*, *Conexibacter*, *Jatrophihabitans*, and fungal genera of *Mollisia*. *A. bacterium* and *A. bacterium* CU2 were identified as potential antagonistic agents. Functionally, chemoheterotrophy and aerobic chemoheterotrophy were the dominant bacterial trophic modes while saprotroph was the dominant fungal trophic mode. Between symptomatic trees and asymptomatic trees, the bacterial community differed in the two stem regions while the fungal community was distinct in each region. At function level, the bacterial community differed in lower stem and fungal community differed in upper stem of symptomatic and asymptomatic trees. *Heterobasidion* infection enlarged the network scale and decreased the network microbial stability in Norway spruce trees. Negative correlations dominated the *Heterobasidion*-centered network, where bacterial ASVs and fungal ASVs dominated negative correlations to the lower stem and upper stem, respectively.

## Data Availability

The data sets generated and analyzed during this study are available in the NCBI SRA 621 database number PRJNA1107117 for bacteria and under project number 622 PRJNA1107041 for fungi.

## Footnotes

1
http://ccb.jhu.edu/software/FLASH/

2
https://www.arb-silva.de/

3
https://unite.ut.ee/

4
https://github.com/torognes/vsearch
